# Neural mechanisms of auditory categorization: from across brain areas to within local microcircuits

**DOI:** 10.3389/fnins.2014.00161

**Published:** 2014-06-17

**Authors:** Joji Tsunada, Yale E. Cohen

**Affiliations:** ^1^Department of Otorhinolaryngology-Head and Neck Surgery, Perelman School of Medicine, University of PennsylvaniaPhiladelphia, PA, USA; ^2^Department of Neuroscience, University of PennsylvaniaPhiladelphia, PA, USA; ^3^Department of Bioengineering, University of PennsylvaniaPhiladelphia, PA, USA

**Keywords:** auditory category, ventral auditory pathway, speech sound, vocalization, pyramidal neuron, interneuron

## Abstract

Categorization enables listeners to efficiently encode and respond to auditory stimuli. Behavioral evidence for auditory categorization has been well documented across a broad range of human and non-human animal species. Moreover, neural correlates of auditory categorization have been documented in a variety of different brain regions in the ventral auditory pathway, which is thought to underlie auditory-object processing and auditory perception. Here, we review and discuss how neural representations of auditory categories are transformed across different scales of neural organization in the ventral auditory pathway: from across different brain areas to within local microcircuits. We propose different neural transformations across different scales of neural organization in auditory categorization. Along the ascending auditory system in the ventral pathway, there is a progression in the encoding of categories from simple acoustic categories to categories for abstract information. On the other hand, in local microcircuits, different classes of neurons differentially compute categorical information.

## Introduction

Auditory categorization is a computational process in which sounds are classified and grouped based on their acoustic features and other types of information (e.g., semantic knowledge about the sounds). For example, when we hear the word “Hello” from different speakers, we can categorize the gender of each speaker based on the pitch of the speaker's voice. On the other hand, in order to analyze the linguistic content transmitted by speech sounds, we can ignore the unique pitch, timbre etc. of each speaker and categorize the sound into the distinct word category “Hello.” Thus, auditory categorization enables humans and non-human animals to extract, manipulate, and efficiently respond to sounds (Miller et al., 2002, 2003; Russ et al., 2007; Freedman and Miller, 2008; Miller and Cohen, 2010).

A specific type of categorization is called “categorical perception” (Liberman et al., 1967; Kuhl and Miller, 1975, 1978; Kuhl and Padden, 1982, 1983; Kluender et al., 1987; Pastore et al., 1990; Lotto et al., 1997; Sinnott and Brown, 1997; Holt and Lotto, 2010). The primary characteristic of categorical perception is that the perception of a sound does not smoothly vary with changes in its acoustic features. That is, in certain situations, small changes in the physical properties of an acoustic stimulus can cause large changes in a listener's perception of a sound. In other situations, large changes can cause no change in perception. The stimuli, which cause these large changes in perception, straddle the boundary between categories. For example, when we hear a continuum of smoothly varying speech sounds (i.e., a continuum of morphed stimuli between the phoneme prototypes “ba” and “da”), we experience a discrete change in perception. Specifically, a small change in the features of a sound near the middle of this continuum (i.e., at the category boundary between a listener's perception of “ba” and “da”) will cause a large change in a listener's perceptual report. In contrast, when that same small change occurs at one of the ends of the continuum, there is little effect on the listener's report.

Even though some perceptual categories have sharp boundaries, the locations of the boundary are somewhat malleable. For instance, the perception of a phoneme can be influenced by the phonemes that come before it. When morphed stimuli, which are made from the prototypes “da” and “ga,” are preceded by presentations of “al” or “ar,” the perceptual boundary between the two prototypes shifts (Mann, 1980). Specifically, listeners' reports are biased toward reporting the morphed stimuli as “da” when it is preceded by “ar.” When this morphed stimulus is preceded by “al,” listeners are biased toward reporting the morphed stimulus as “ga.”

Categories are not only formed based on the perceptual features of stimuli but also on more “abstract” types of information. An abstract category is one in which a group of arbitrary stimuli are linked together as a category based on some shared features, a common functional characteristic, semantic information, or acquired knowledge. For instance, a combination of physical characteristics and knowledge about their reproductive processes puts dogs, cats, and killer whales into one category (“mammals”), but birds into a separate category. However, if we use different criteria to form a category of “pets,” dogs, cats, and birds would be members of this “pet” category but not killer whales.

Behavioral responses to auditory communication signals (i.e., species-specific vocalizations) also provide evidence for abstract categorization. One example is the categorization of food-related species-specific vocalizations by rhesus monkeys (Hauser and Marler, 1993a,b; Hauser, 1998; Gifford et al., 2003). In rhesus monkeys, a vocalization called a “harmonic arch” transmits information about the discovery of rare, high-quality food. A different vocalization called a “warble” also transmits the same type of information: the discovery of rare, high-quality food. Importantly, whereas both harmonic arches and warbles transmit the same type of information, they have distinct spectrotemporal properties. Nevertheless, rhesus monkeys' responses to those vocalizations indicate that monkeys categorize these two calls based on their transmitted information and not their acoustic features. In another example, Diana monkeys form abstract-categorical representations for predator-specific alarm calls independent of the species generating the signal. Diana monkeys categorize and respond similarly to alarm calls that signify the presence of a leopard, regardless of whether the alarm calls are elicited from a Diana monkey or a crested guinea fowl (Zuberbuhler and Seyfarth, 1997; Züberbuhler, 2000a,b). Similarly, Diana monkeys show similar categorical-responses to eagle alarm calls that can be elicited from other Diana monkeys or from putty-nose monkeys (Eckardt and Zuberbuhler, 2004).

In order to better understand the mechanisms that underlie auditory categorization, it is essential to examine how neural representations of auditory categories are formed and transformed across different scales of neural organization: from across different brain areas to within local microcircuits. In this review, we discuss the representation of auditory categories in different cortical regions of the ventral auditory pathway; the hierarchical processing of categorical information along the ventral pathway; and the differential role that excitatory pyramidal neurons and inhibitory interneurons (i.e., different neuron classes) contribute to these categorical computations.

**Figure 1:**
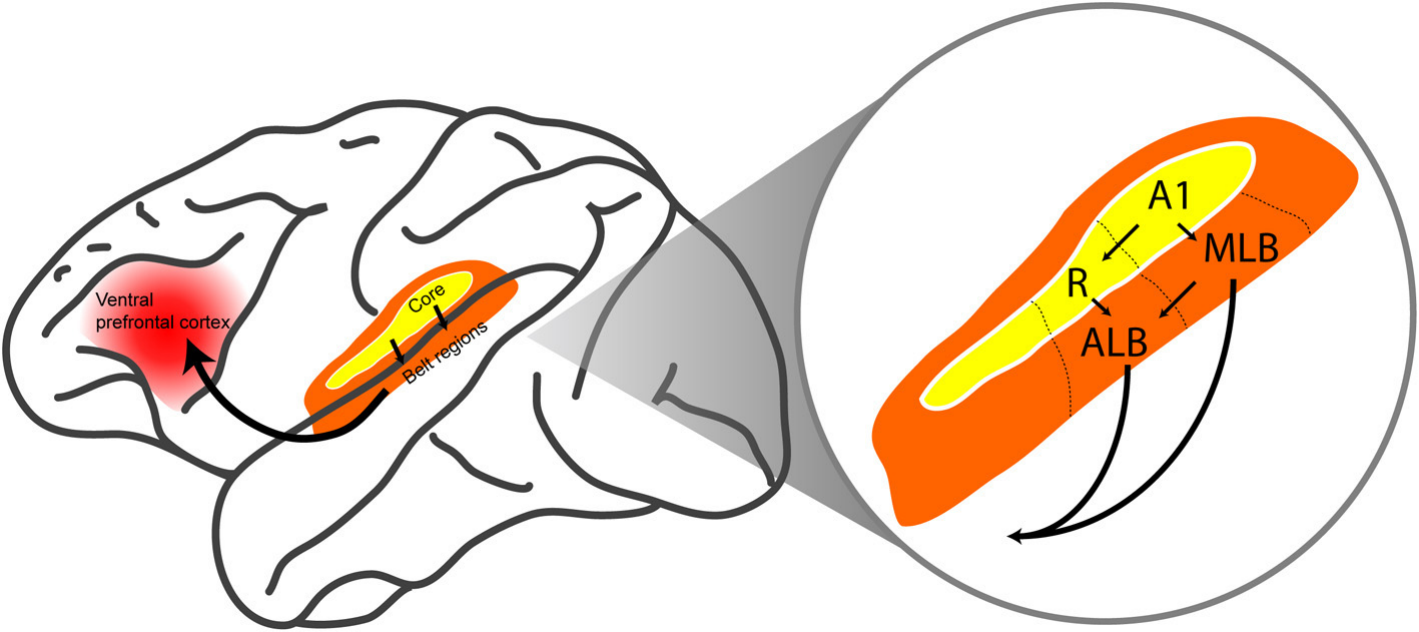
**The ventral auditory pathway in the monkey brain**. The ventral auditory pathway begins in core auditory cortex (in particular, the primary auditory cortex A1 and the rostral field R). The pathway continues into the middle-lateral (MLB) and anterolateral (ALB) belt regions, which project directly and indirectly to the ventral prefrontal cortex. Arrows indicate feedforward projections. The figure is modified, with permission, from Hackett et al. (1998) and Romanski et al. (1999a).

The ventral pathway is targeted because neural computations in this pathway are thought to underlie sound perception, which is critically related to auditory categorization and auditory scene analysis (Rauschecker and Scott, 2009; Romanski and Averbeck, 2009; Bizley and Cohen, 2013). The ventral auditory pathway begins in the core auditory cortex (in particular, the primary auditory cortex and the rostral field R) and continues into the anterolateral and middle-lateral belt regions. These belt regions then project either directly or indirectly to the ventral prefrontal cortex (Figure 1) (Hackett et al., 1998; Rauschecker, 1998; Kaas and Hackett, 1999, 2000; Kaas et al., 1999; Romanski et al., 1999a,b; Rauschecker and Tian, 2000; Rauschecker and Scott, 2009; Romanski and Averbeck, 2009; Recanzone and Cohen, 2010; Bizley and Cohen, 2013).

## Neural transformations across cortical areas in the ventral auditory pathway

In this section, we discuss how auditory categories are processed in the ventral auditory pathway. More specifically, we review the representation of auditory categories across different regions in the ventral auditory pathway and then discuss the hierarchical processing of categorical information in the ventral auditory pathway.

Before we continue, it is important to define the concept of a “neural correlate of categorization.” One simple definition is the following: a neural response is “categorical” when the responses are invariant to the stimuli that belong to the same category. In practice, neuroimaging techniques define “categorical” responses as equivalent activations of distinct brain regions by within-category stimuli and the equivalent activation of different brain regions by stimulus exemplars from a second category (Binder et al., 2000; Altmann et al., 2007; Doehrmann et al., 2008; Leaver and Rauschecker, 2010). At the level of single neurons, a neuron is said to be “categorical” if its firing rate is invariant to different members of one category and if it has a second level of (invariant) responsivity to stimulus exemplars from a second category (Freedman et al., 2001; Tsunada et al., 2011). The specific mechanisms that underlie the creation of category sensitive neurons are not known. However, presumably, they rely on the computations that mediate stimulus invariance in neural selectivity and perception (Logothetis and Sheinberg, 1996; Holt and Lotto, 2010; Dicarlo et al., 2012). Moreover, because animals can form a wide range of categories based on individual experiences, a degree of learning and plasticity must be involved in the creation of *de-novo* category selective responses (Freedman et al., 2001; Freedman and Assad, 2006). Indeed, when monkeys were trained to categorize stimuli with different category boundaries, boundaries for categorical responses in some brain areas (e.g., the prefrontal and parietal cortices) also changed (Freedman et al., 2001; Freedman and Assad, 2006).

### How do different cortical areas in the ventral auditory pathway similarly or differentially represent categorical information?

It is well known that neurons become increasingly sensitive to more complex stimuli and abstract information between the beginning stages of the ventral auditory pathway (i.e., the core) and the latter stages (e.g., the ventral prefrontal cortex). For example, neurons in the core auditory cortex are more sharply tuned for tone bursts than neurons in the lateral belt (Rauschecker et al., 1995), whereas lateral-belt neurons are more sensitive to the spectrotemporal properties of complex sounds, such as vocalizations (Rauschecker et al., 1995; Tian and Rauschecker, 2004). Furthermore, beyond the auditory cortex, the ventral prefrontal cortex not only encodes complex sounds (Averbeck and Romanski, 2004; Cohen et al., 2007; Russ et al., 2008a; Miller and Cohen, 2010) but also has a critical role for attention and memory-related cognitive functions (e.g., memory retrieval) which are critical for abstract categorization (Goldman-Rakic, 1995; Miller, 2000; Miller and Cohen, 2001; Miller et al., 2002, 2003; Gold and Shadlen, 2007; Osada et al., 2008; Cohen et al., 2009; Plakke et al., 2013a,b,c; Poremba et al., 2013).

These observations are consistent with the idea that there is a progression of category-information processing along the ventral auditory pathway: brain regions become increasingly sensitive to more complex types of categories. More specifically, it appears that neurons in core auditory cortex may encode categories for simple sounds, whereas neurons in the belt regions and the ventral prefrontal cortex may encode categories for more complex sounds and abstract information.

Indeed, neural correlates of auditory categorization can be seen in the core auditory cortex for simple frequency contours (Ohl et al., 2001; Selezneva et al., 2006). For example, in a study by Selezneva and colleagues, monkeys categorized the direction of a frequency contour of tone-burst sequences as either “increasing” or “decreasing” while neural activity was recorded from the primary auditory cortex. Selezneva et al. found that these core neurons encoded the sequence direction independent of its specific frequency content: that is, a core neuron responded similarly to a decreasing sequence from 1 to 0.5 kHz as it did to a decreasing sequence from 6 to 3 kHz. In a second study, Ohl et al. demonstrated that categorical representations need not be represented in the firing rates of single neurons but, instead, can be encoded in the dynamic firing patterns of a neural population. Thus, even in the earliest stage of the ventral auditory pathway, there is evidence for neural categorization.

Although the core auditory cortex processes categorical information for simple auditory stimuli (e.g., the direction of frequency changes of pure tones), studies using more complex sounds, such as human-speech sounds, have shown that core neurons primarily encode the acoustic features that compose these complex sounds but do not encode their category membership (Liebenthal et al., 2005; Steinschneider et al., 2005; Obleser et al., 2007; Engineer et al., 2008, 2013; Mesgarani et al., 2008, 2014; Nourski et al., 2009; Steinschneider, 2013). That is, the categorization of complex sounds requires not only analyses at the level of the acoustic feature but also subsequent computations that integrate the analyzed features into a perceptual representation, which is then subject to a categorization process. For example, distributed and temporally dynamic neural responses in individual core neurons can represent different acoustic features of speech sounds (Schreiner, 1998; Steinschneider et al., 2003; Engineer et al., 2008; Mesgarani et al., 2008, 2014), but the categorization of the speech sounds requires classifying the activation pattern across the entire population of core neurons.

**Figure 2:**
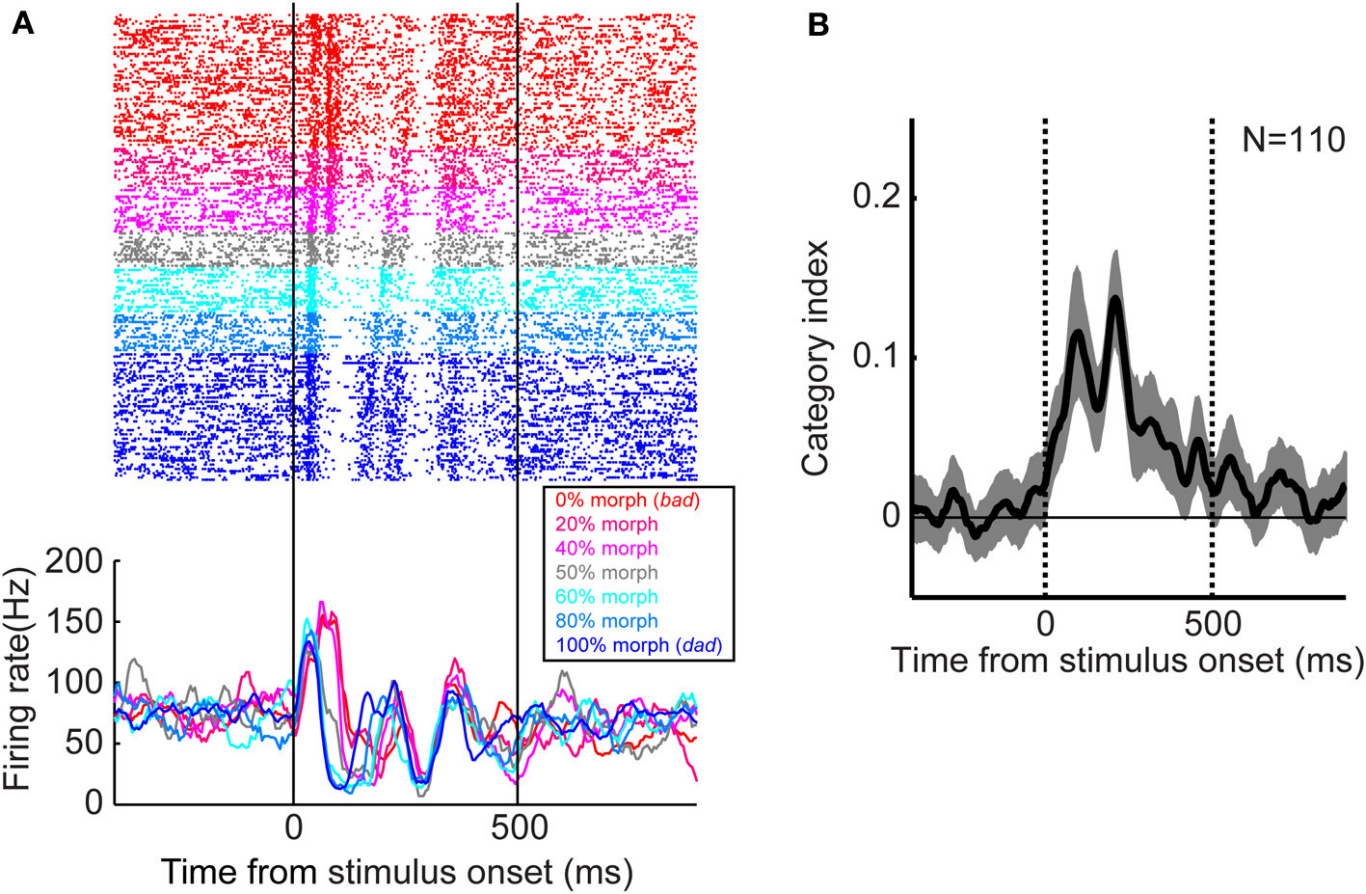
**Categorical neural activity in the monkey lateral belt during categorization of speech sounds. (A)** An example of the activity of a lateral belt neuron. The speech sounds were two human-speech sounds (“bad” and “dad”) and their morphs. Neural activity is color-coded by morphing percentage of the stimulus as shown in the legend. The raster plots and histograms are aligned relative to onset of the stimulus. **(B)** Temporal dynamics of the category index at the population level. Category-index values >0 indicate that neurons categorically represent speech sounds (Freedman et al., 2001; Tsunada et al., 2011). The thick line represents the mean value and the shaded area represents the bootstrapped 95%-confidence intervals of the mean. The two vertical lines indicate stimulus onset and offset, respectively, whereas the horizontal line indicates a category-index value of 0. The figure is adopted, with permission, from Tsunada et al. (2011).

Categorical representations of speech sounds at the level of the single neuron or local populations of neurons appear to occur at the next stage of auditory processing in the ventral auditory pathway, the lateral-belt regions. Several recent studies have noted that neural activity in the monkey lateral-belt and human superior temporal gyrus encodes speech-sound categories (Chang et al., 2010; Steinschneider et al., 2011; Tsunada et al., 2011; Steinschneider, 2013). For example, our group found that, when monkeys categorized two prototypes of speech sounds (“bad” and “dad”) and their morphed versions, neural activity in the lateral belt discretely changed at the category boundary, suggesting that these neurons encoded the auditory category rather than smoothly varying acoustic features (Figure 2).

Human-neuroimaging studies have also found that the superior temporal sulcus is categorically activated by speech sounds, relative to other sounds (Binder et al., 2000; Leaver and Rauschecker, 2010). Specifically, the superior temporal sulcus was activated more by speech sounds than by frequency-modulated tones (Binder et al., 2000) or by other sounds including bird songs and animal vocalizations (Leaver and Rauschecker, 2010). Furthermore, activity in the superior temporal sulcus did not simply reflect the acoustic properties of speech sounds but, instead, represented the perception of speech (Mottonen et al., 2006; Desai et al., 2008).

Additionally, studies with other complex stimuli provide further evidence for the categorical encoding of complex sounds in the human non-primary auditory cortex, including superior temporal gyrus and sulcus, but not in the core auditory cortex (Altmann et al., 2007; Doehrmann et al., 2008; Leaver and Rauschecker, 2010). These studies found that complex sound categories were represented in spatially distinct and widely distributed sub-regions within the superior temporal gyrus and sulcus (Obleser et al., 2006, 2010; Engel et al., 2009; Staeren et al., 2009; Chang et al., 2010; Leaver and Rauschecker, 2010; Giordano et al., 2013). For example, distinct regions of the superior temporal gyrus and sulcus are selectively activated by musical-instrument sounds (Leaver and Rauschecker, 2010), tool sounds (Doehrmann et al., 2008), and human-speech sounds (Belin et al., 2000; Binder et al., 2000; Warren et al., 2006); whereas the anterior part of the superior temporal gyrus and sulcus is preferentially activated by the passive listening of conspecific vocalizations than other vocalizations (Fecteau et al., 2004). Similar findings for con-specific vocalizations have been obtained in the monkey auditory cortex (Petkov et al., 2008; Perrodin et al., 2011). Consistent with these findings, neuropsychological studies have shown that human patients with damage in the temporal cortex have deficits in voice recognition and discrimination (i.e., phonagnosia Van Lancker and Canter, 1982; Van Lancker et al., 1988; Goll et al., 2010). Thus, hierarchically higher regions in the auditory cortex encode complex-sound categories in spatially distinct (i.e., modular) and widely distributed sub-regions.

Moreover, recent studies posit that the sub-regions in the non-primary auditory cortex process categorical information in a hierarchical manner (Warren et al., 2006). A recent meta-analysis of human speech-processing studies suggests that a hierarchical organization of speech processing exists within the superior temporal gyrus: the middle superior temporal gyrus is sensitive to phonemes; anterior superior temporal gyrus to words; and the most anterior locations to short phrases (Dewitt and Rauschecker, 2012; Rauschecker, 2012). Additionally, a different hierarchical processing of speech sounds in the superior temporal sulcus has also been articulated: the posterior superior temporal sulcus is preferentially sensitive for newly acquired sound categories, whereas the middle and anterior superior temporal sulci are more responsive to familiar sound categories (Liebenthal et al., 2005, 2010). Thus, within different areas of the non-primary auditory cortex, multiple and parallel processing may progress during auditory categorization.

**Figure 3:**
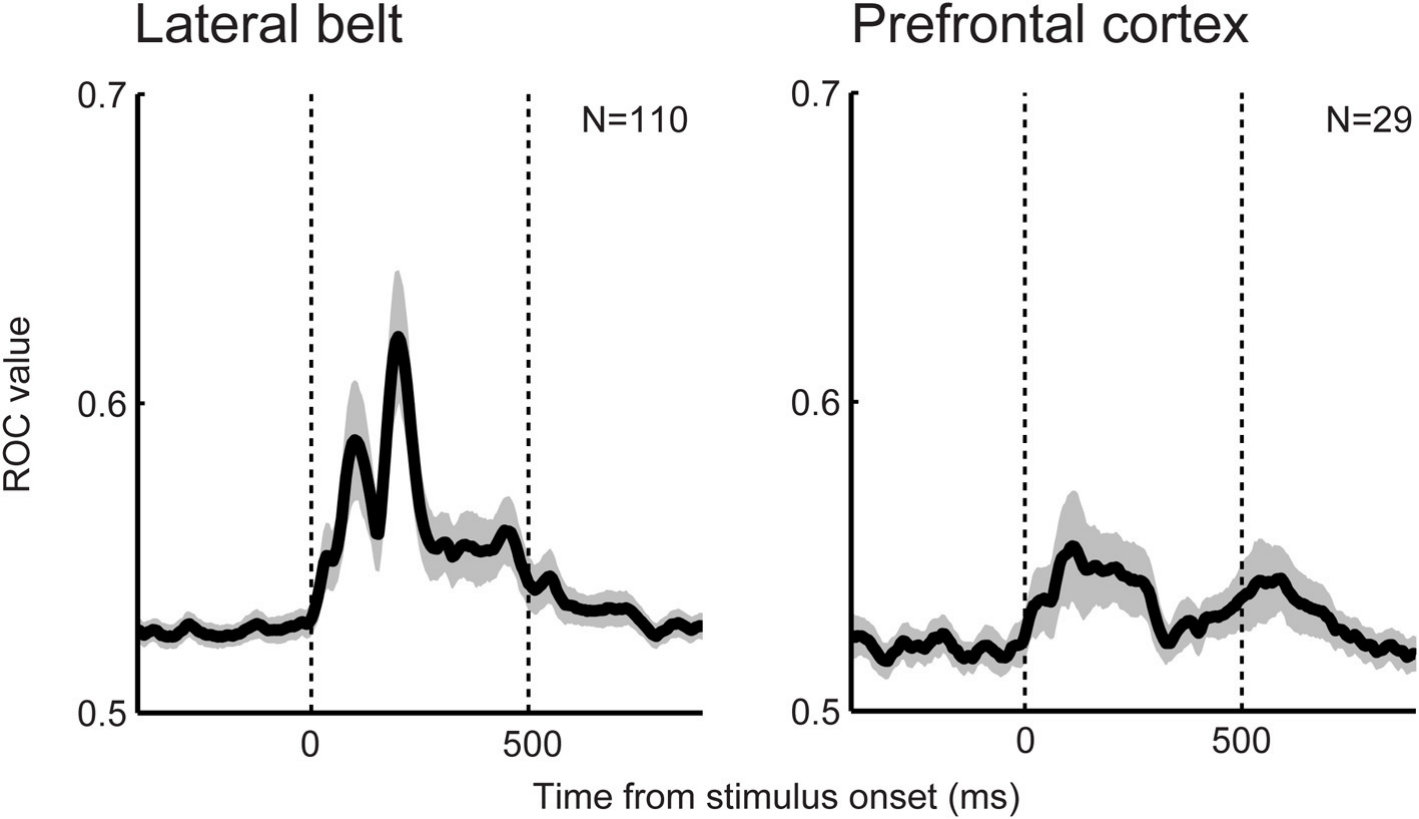
**Category sensitivity for speech sounds in the prefrontal cortex (right) is weaker than that in the lateral belt (left)**. Temporal dynamics of the category sensitivity at the population level are shown. Category sensitivity was calculated using a receiver-operating-characteristic (ROC) analysis (Green and Swets, 1966; Tsunada et al., 2012). Larger ROC values indicate better differentiation between the two categories.

Beyond the auditory cortex, do latter processing stages (e.g., the monkey ventral prefrontal cortex and human inferior frontal cortex) process categories for even more complex sounds? A re-examination of previous findings from our lab (Russ et al., 2008b; Tsunada et al., 2011) indicated important differences in neural categorization between the lateral belt and the ventral prefrontal cortex (Figure 3). We found that, at the population level, the category sensitivity for speech sounds in the prefrontal cortex was weaker than that in the lateral belt although neural activity in the prefrontal cortex transmitted a significant amount of categorical information. Consistent with this finding, a human-neuroimaging study also found that neural activity in the superior temporal gyrus is better correlated with a listener's ability to discriminate between speech sounds than the activity in the inferior prefrontal cortex (Binder et al., 2004). Because complex sounds, including speech sounds, are substantially processed in the non-primary auditory cortex as discussed above, the prefrontal cortex may not represent, relative to the auditory cortex, a higher level of auditory perceptual-feature categorization.

Instead, the prefrontal cortex may be more sensitive to categories that are formed based on the abstract information that is transmitted by sounds. For example, the human inferior prefrontal cortex may encode categories for abstract information like emotional valence of a speaker's voice (Fecteau et al., 2005). Furthermore, human electroencephalography and neuroimaging studies have also revealed that the inferior prefrontal cortex plays a key role in the categorization of semantic information of multisensory stimuli (Werner and Noppeney, 2010; Joassin et al., 2011; Hu et al., 2012): Joassin et al. showed that the inferior prefrontal cortex contains multisensory category representations of gender that is derived from a speaker's voice and from visual images of a person's face.

Similarly, the monkey ventral prefrontal cortex encodes abstract categories. We have found that neurons in the ventral prefrontal cortex represent categories for food-related calls based on the transmitted information (e.g., high quality food vs. low quality food) (Gifford et al., 2005; Cohen et al., 2006). A more recent study found that neural activity in the monkey prefrontal cortex categorically represents the number of auditory stimuli (Nieder, 2012). Thus, along the ascending auditory system in the ventral auditory pathway, cortical areas encode categories for more complex stimuli and more abstract information.

## Neural transformations within local microcircuits

In this section, we discuss how the categorical information represented in each cortical area of the ventral auditory pathway is computed within local microcircuits. First, we briefly review the cortical microcircuit. Next, we focus on the role that two main cell classes of neurons in cortical microcircuits (i.e., excitatory pyramidal neurons and inhibitory interneurons) and discuss how different classes of neurons process categorical information.

### How do different classes of neurons in local microcircuits process categorical information?

A cortical microcircuit can be defined as a functional unit that processes inputs and generates outputs by dynamic and local interactions of excitatory pyramidal neurons and inhibitory interneurons (Merchant et al., 2012). Consequently, pyramidal neurons and interneurons are considered to be the main elements of microcircuits. Pyramidal neurons, which consist ~70–90% of cortical neurons, provide excitatory-outputs locally (i.e., within a cortical area) and across brain areas (Markham et al., 2004). On the other hand, interneurons, which consist small portion of cortical neurons (~10–30%), provide mainly inhibitory-outputs to surrounding pyramidal neurons and other interneurons (Markham et al., 2004).

From a physiological perspective, pyramidal neurons and interneurons can be classified based on the waveform of their action potentials (Mountcastle et al., 1969; McCormick et al., 1985; Kawaguchi and Kubota, 1993, 1997; Kawaguchi and Kondo, 2002; Markham et al., 2004; González-Burgos et al., 2005). More specifically, the waveforms of pyramidal neurons tend to be broader and slower than those seen in the most interneurons. Using this classification, several extracellular-recording studies have been able to elucidate roles of pyramidal neurons and interneurons for visual working memory in the prefrontal cortex (Wilson et al., 1994; Rao et al., 1999; Constantinidis and Goldman-Rakic, 2002; Diester and Nieder, 2008; Hussar and Pasternak, 2012), visual attention in V4 (Mitchell et al., 2007), visual perceptual decision-making in the frontal eye field (Ding and Gold, 2011), motor control in the motor and premotor cortices (Isomura et al., 2009; Kaufman et al., 2010), and auditory processing during the passive listening in the auditory cortex (Atencio and Schreiner, 2008; Sakata and Harris, 2009; Ogawa et al., 2011). Interestingly, most of these studies showed differential roles in pyramidal neurons and interneurons.

**Figure 4:**
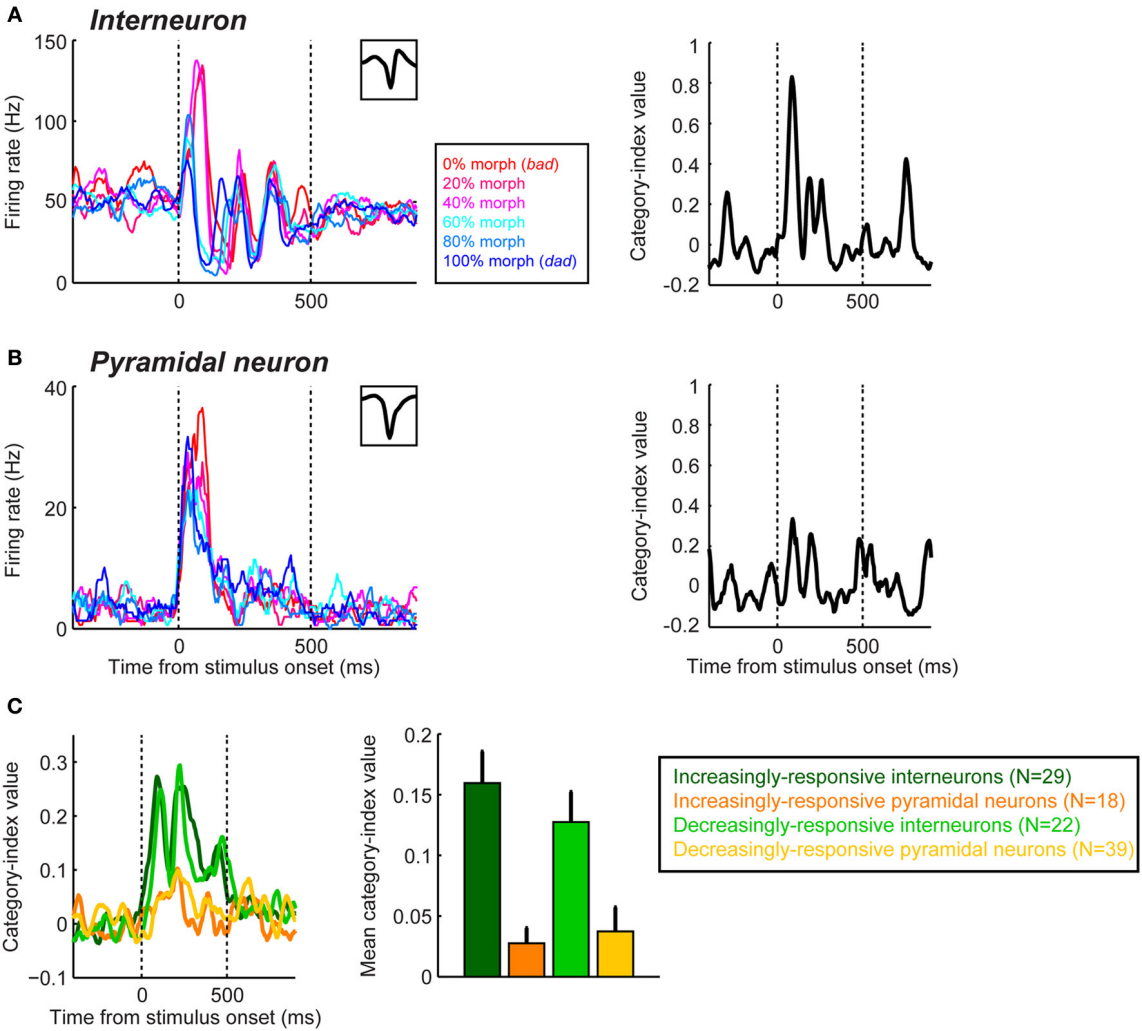
**Category sensitivity in interneurons is greater than that seen in pyramidal neurons during categorization of speech sounds in the auditory cortex**. The plots in the left column of panel **(A,B)** show the mean firing rates of an interneuron **(A)** and a pyramidal neuron **(B)** as a function of time and the stimulus presented. The stimuli were two human-speech sounds (“bad” and “dad”) and their morphs. Neural activity is color-coded by morphing percentage of the stimulus as shown in the legend. The inset in the upper graph of each plot shows the neuron's spike-waveform. The right column shows each neuron's category-index values as a function of time. For all of the panels, the two vertical dotted lines indicate stimulus onset and offset, respectively. **(C)** Population results of category index. The temporal profile (left panel) and mean (right) of the category index during the stimulus presentation are shown. Putative interneurons and pyramidal neurons were further classified as either “increasingly responsive” or “decreasingly responsive” based on their auditory-evoked responses. Error bars represent bootstrapped 95% confidence intervals of the mean. The figure is adopted, with permission, from Tsunada et al. (2012).

Recently, using differences in the waveform of extracellularly-recorded neurons, we found that putative pyramidal neurons and interneurons in the lateral belt differentially encode and represent auditory categories (Tsunada et al., 2012). Specifically, we found that interneurons, on average, are more sensitive for auditory-category information than pyramidal neurons, although both neuron classes reliably encode category information (Figure 4).

Unfortunately, to our knowledge, there have not been other auditory-category studies that have examined the relative category sensitivity of pyramidal neurons vs. interneurons. However, a comparable visual-categorization study on numerosity in the prefrontal cortex (Diester and Nieder, 2008) provides an opportunity to compare results across studies. Unlike our finding, Diester and Nieder found greater category sensitivity for putative pyramidal neurons than for putative interneurons.

The bases for these different sets of findings are unclear. However, three non-exclusive possibilities may underlie these differences. One possibility may relate to differences in the local-connectivity patterns and interactions between pyramidal neurons and interneurons across cortical areas (Wilson et al., 1994; Constantinidis and Goldman-Rakic, 2002; Diester and Nieder, 2008; Kätzel et al., 2010; Tsunada et al., 2012). Indeed, in the prefrontal cortex, simultaneously recorded (and, hence, nearby) pyramidal neurons and interneurons have different category preferences (Diester and Nieder, 2008). In contrast, in the auditory cortex, simultaneously recorded pairs of pyramidal neurons and interneurons have similar category preferences (Tsunada et al., 2012). Thus, there may be different mechanisms for shaping category sensitivity across cortical areas. Second, the nature of the categorization task may also affect, in part, the category sensitivity of pyramidal neurons and interneurons: our task was a relatively simple task requiring the categorization of speech sounds based primarily on perceptual similarity, whereas Diester and Nieder's study required a more abstract categorization of numerosity. Finally, the third possibility relates to differences between stimulus dynamics: the visual stimuli in the Diester and Nieder's study were static stimuli, whereas our speech sounds had a rich spectrotemporal dynamic structure. To categorize dynamic stimuli, the moment-by-moment features of stimuli need to be quickly categorized. Thus, the greater category sensitivity of interneurons along with their well-known inhibitory influence on pyramidal neurons (Hefti and Smith, 2003; Wehr and Zador, 2003; Atencio and Schreiner, 2008; Fino and Yuste, 2011; Isaacson and Scanziani, 2011; Packer and Yuste, 2011; Zhang et al., 2011) may underlie the neural computations needed to create categorical representations of dynamic stimuli in the auditory cortex.

## Conclusions and future directions

Different neural transformations across different scales of neural organization progress during auditory categorization. Along the ascending auditory system in the ventral pathway, there is a progression in the encoding of categories from simple acoustic categories to categories representing abstract information. On the other hand, in local microcircuits within a cortical area, different classes of neurons, pyramidal neurons and interneurons, differentially compute categorical information. The computation is likely dependent upon the functional organization of the cortical area and dynamics of stimuli.

Despite several advances in our understanding of neural mechanism of auditory categorization, there still remain many important questions to be addressed. For example, it is poorly understood how bottom-up inputs from hierarchically lower areas, top-down feedback from higher areas, and local computations interact to form neural representations of auditory categories. Answering this question will provide a more thorough understanding of the information flow in the ventral auditory pathway. Another important question to be tested is what neural circuit mechanisms produce different category sensitivity between pyramidal neurons and interneurons, and functional roles of pyramidal neurons and interneurons in auditory categorization. Relevant to this question, the role that cortical laminae (another key element of local microcircuitry) play in auditory categorization should be also tested. Recent advances in experimental and analysis techniques should enable us to clarify the functional role of different classes of neurons in auditory categorization (Letzkus et al., 2011; Znamenskiy and Zador, 2013) and also test neural categorization across cortical layers (Lakatos et al., 2008; Takeuchi et al., 2011), providing further insights for neural computations for auditory categorization within local microcircuits.

### Conflict of interest statement

The authors declare that the research was conducted in the absence of any commercial or financial relationships that could be construed as a potential conflict of interest.
